# Developing a fall prevention program in an inpatient spinal cord injury rehabilitation unit: A participatory action research study

**DOI:** 10.1371/journal.pone.0304320

**Published:** 2024-07-10

**Authors:** Shoeleh Rahimi, Hamid Reza Khankeh, Abbas Ebadi, Batol Mohammadian, Mohammad Eghbali, Fatemeh habibzadeh

**Affiliations:** 1 PhD in Nursing, University of Social Welfare and Rehabilitation Sciences (USWR), Tehran, IR Iran; 2 Nurse of Javadalaimeh Hospital in Kharameh, Shiraz University of Medical Sciences, Shiraz, Fars, Iran; 3 Health in Emergency and Disaster Research Center, Social Health Research Institute, University of Social Welfare and Rehabilitation Sciences, Tehran, Iran; 4 Behavioral Sciences Research Center, Life Style Institute, Baqiyatallah University of Medical Sciences, Tehran, Iran; 5 Faculty of Nursing, Baqiyatallah University of Medical Sciences, Teheran, Iran; 6 Department of Operating Room, School of Allied Medical Sciences, Gonabad University of Medical Sciences, Gonabad, Iran; 7 Department of Nursing, school of Nursing and Midwifery, Torbat Heydariyeh University of Medical Sciences, Torbat Heydariyeh, Iran; 8 MSC of Critical Care Nursing Tehran University of Medical Science, Tehran, Iran; Iran University of Medical Sciences, ISLAMIC REPUBLIC OF IRAN

## Abstract

**Importance:**

Spinal cord injury is a lifelong disability necessitating early management of falls during inpatient admissions. However, there is a paucity of research on fall prevention and management in Spinal cord injury rehabilitation.

**Objective(s):**

This study aimed at developing a fall prevention program in an inpatient rehabilitation Spinal cord injury unit.

**Method:**

A participatory action research approach utilizing a before-and-after, mixed-method design was employed for this study. The study was performed at Rofaydeh Rehabilitation Hospital in Tehran, Iran, from 2021 to 2022. the study implemented Cohen’s four-stage model, encompassing the design of a change program, action, observation and evaluation, and feedback stages. A purposeful sampling method was utilized to select 19 nurses and members of the rehabilitation team from the hospital, ensuring maximum diversity. Data collection involved semi-structured interviews, focus groups, and a checklist for fall prevention measures. Qualitative content analysis, alongside descriptive (frequency, mean, and standard deviation) and inferential statistics (paired t-tests and Chi-square tests), were employed for data analysis. The study adhered to COREQ guidelines.

**Results:**

Falls were most common among patients aged fifty years or older (P = 0.026). Throughout the study period, men were more likely to experience falls than women (P = 0.01). Preventive interventions have led to significant improvements in indicators of patient monitoring and care, patient education, and environmental safety, as demonstrated by a paired-sample t-test (P<0.001). Moreover, factors contributing to patients’ falls included "shortcomings in fall prevention policies" and "lack of knowledge and participation among patients and caregivers." Changes implemented in the Spinal Cord Injury unit involved enhancing interprofessional interactions, conducting educational workshops for patients and their companions, and identifying high-risk patients. These findings indicate a significant decrease in the incidence of falls following the intervention (P = 0.02).

**Conclusions:**

The study found that a multifaceted intervention can increase knowledge about fall risks and substantially reduce both falls and associated minor injuries.

## Background

Falls during a hospital stay pose a significant threat to patient safety [1]. A patient fall is defined as an the uncontrolled and sudden descent of a patient’s body to the floor, with or without injury risk [2]. Patients with spinal cord injury (SCI) often experience motor and sensory changes that increase their susceptibility to falls [3], especially as they navigate their limitations and acquire new skills during inpatient rehabilitation [2]. Since SCI is a permanent disability, it is crucial to address fall prevention early during inpatient admissions reported fall rates for inpatient SCI in neurorehabilitation wards can be as high as 24%, emphasizing the importance of implementing fall prevention measures during inpatient rehabilitation. Understanding the dynamics of falls during this stage of recovery is essential [4].

Fall-related injuries place a considerable economic burden on patients and the healthcare system.

Moreover, they can result in pain, bruises, cuts, fractures, and loss of consciousness upon falling [5, 6]. Individuals who experience falls may develop a condition known as "post-fall syndrome," marked by a fear of falling, reduced physical activity and independence [7], decreased involvement in rehabilitation programs [5], prolonged treatment duration and hospital stay, ultimately disrupting rehabilitation goals [8]. Therefore, fall prevention should be integrated throughout the entire inpatient admission process [4].

Previous studies have examined multifactorial interventions for fall prevention, including assessing fall risk and mitigating these risks through education, environmental modifications, and the utilization of various technologies such as continuous video monitoring, smart alarms, and wearable sensors [9]. Despite the critical importance of fall prevention in inpatient SCI patients, prior research has identified several barriers to the implementation of fall prevention strategies. These include inadequacies in hospital policies, insufficient patient monitoring, issues related to patients’ gait performance and cognitive impairment, environmental hazards, and staff-related challenges such as lack of training and commitment to implementing fall prevention policies [10–12]. Conversely, evidence suggests that the current policies and procedures for preventing falls in inpatient SCI patients at rehabilitation hospitals closely resemble those implemented in general hospitals and community settings [13–15]. This similarity poses challenges for nurses and other clinical staff when implementing fall prevention approaches specific to SCI rehabilitation [16]. For example, there is a requirement for a specialized risk assessment scale to identify limitations in SCI patients and assess the risk of falls within this population [17, 18]. Furthermore, individuals with SCI have reported receiving inadequate education on fall prevention and practical strategies during rehabilitation [16].

Understanding the unique circumstances surrounding falls during SCI rehabilitation can facilitate the development of targeted and individualized fall prevention programs early in the rehabilitation process [4]. Action research stands out as one of the most effective research methods for addressing issues within care settings. It is a systematic approach that enables participants to articulate their research needs and devise strategies to tackle these needs. Action research proves particularly effective when both managers and stakeholders actively engage in the change process, tailoring interventions to the specific conditions and culture of the organization’s setting [19].

In Thailand, a study utilized this method to craft a fall prevention program for elderly patients. The outcomes demonstrated a decrease in fall incidence, enhancements in fall prevention behaviors, and high participant satisfaction with the program [9]. Likewise, in Australia, an action research study aimed to deepen understanding of fall prevention among elderly patients with dementia [20]. Additionally, this approach was employed to design a fall prevention program for multiple sclerosis (MS) patients in England [21].

The perspective of healthcare workers on fall prevention can significantly influence their willingness to engage in specific preventive interventions. To formulate acceptable and practical fall prevention strategies, the views of stakeholders must also be considered [22]. Therefore, the current study employed a participatory action research approach to identify existing issues in fall prevention through the involvement of relevant stakeholders. Subsequently, with their collaboration, necessary interventions were planned and implemented to reduce fall rates in the SCI unit within an inpatient rehabilitation hospital.

## Materials and methods

### Study design and setting

A mixed-method design, incorporating a before-after approach, was employed within a participatory action research (PAR) framework. The study took place at Rofaydeh Rehabilitation Hospital in Tehran, Iran, spanning from 2021 to 2022. The research focused on the spinal cord injury (SCI) unit within an 80-bed rehabilitation facility. This unit caters to patients aged 16 and above who have experienced traumatic or non-traumatic spinal cord injuries within the past year. Patients typically undergo sub-acute rehabilitation programs, with an average hospitalization period of about four weeks. An interdisciplinary team comprising physicians, nurses, and rehabilitation specialists delivers specialized medical services to these patients.

### Study population and sampling methods

Nursing, rehabilitation team members, and administrative staff were involved in this study, totaling 19 participants across all stages. To ensure diversity in sampling, participants were selected using purposeful sampling, considering various roles, occupational statuses, ages, education levels, and professional experience. Inclusion criteria for staff comprised willingness to cooperate with the research team, a minimum of six months of professional experience in the SCI unit, normal psychological and physical health to participate in interviews, and no history of mental disorders. Participants who were unwilling to continue participating in the study were excluded based on the exclusion criterion.

### Study procedure

In this study, Cohen’s four-stage model, consisting of the following stages, was utilized [23]:

1. **Designing a change program:**
  In the first phase of the study, after introducing the research objectives to the participants and obtaining their consent, the status of fall prevention program in the SCI unit was assessed and the priority areas for fall prevention were determined through collaboration with the staff and departmental officials. Both qualitative methods and quantitative methods were used to identify the main challenges and priorities for fall prevention in the SCI unit. The investigation into the problems of the fall prevention program and potential strategies was based on findings from interviews with participants, focus groups, and quantitative assessments. The program design was informed by interviews and literature reviews. The study adhered to the Consolidated Criteria for Reporting Qualitative Research (COREQ) guidelines.
2. **Action to change the performance:**
  During the implementation stage, participants were briefed about the program during a session. The program was carried out over a span of 4 months, from January to April 2022.
3. **Observation and evaluation:**
  During this stage, issues pertaining to the program’s implementation were identified through participant interviews, observations, and document reviews. The implementation process and potential adjustments were evaluated throughout the program. Efforts were made to address identified problems during program implementation. Program evaluation involved completing adherence checklists for fall prevention measures and comparing them between four months before and after program implementation. Additionally, the number of patient falls was compared between these two time points.
4. **Re -feedback:**
  The feedback from the findings led to the revision of the program and the introduction of new strategies to enhance the change program.

### Data collection

This study employed both quantitative and qualitative approaches simultaneously.

### Qualitative data and analysis

Data collection for this study involved employing in-depth individual interviews, focus groups, and observations to gather information regarding the investigation of problems and the formulation of strategies for fall prevention programs. Interview times were scheduled by participants, and interviews took place in one of the SCI unit rooms. Prior to participation, the purpose of the study was explained to each participant, and written informed consent was obtained.

Semi-structured interviews were conducted to explore participants’ experiences and perceptions of fall prevention. The interviews began with open-ended questions, such as "What factors influence patient falls?", "What do you see as barriers and facilitators to the implementation of fall prevention programs?", "How do you suggest addressing these barriers?", and "What resources are needed to enhance fall prevention programs?" Subsequent questions delved deeper based on participants’ responses, such as "Can you elaborate further?", "Have you had any experiences related to this?", and "Why?" Interviews continued until data saturation was achieved, typically lasting between 45 and 60 minutes each. All interviews were conducted by the first author of the article (Sh.R), who possessed proficiency in qualitative research methods.

Three focus group discussions, each comprising at least six participants, were conducted throughout the study. The first focus group aimed to analyze interviews and identify problems, while subsequent sessions focused on generating potential solutions and discussing the program’s impact. These discussions were scheduled to accommodate stakeholders’ availability across different shifts. All interviews and focus group sessions were audio-recorded and subsequently transcribed verbatim for analysis.

According to the exploratory and descriptive purpose of the study, qualitative content analysis was used to analyze the data using the Lindgren content analysis method (2020) [24]. After transcribing the interviews verbatim, the transcriptions were read to get the general feeling in the de-contextualization stage. Then, the following stages were performed to analyze the data:

- Dividing the transcriptions into summarized semantic units
- Abstracting the summarized semantic units and labeling them using codes
- Classifying the codes based on similarity
- Examining the semantic and conceptual differences of the codes until subcategories formed as indicators of the hidden content of the text
- Finding a meaningful conceptual model of the relationship between subcategories in the re-contextualization stage
- Extracting the main classes

Initial analyzes were performed by the first author (Sh. R) and then the findings were evaluated and finalized by the other authors. Since the researchers did not observe any new concepts after conducting 19 interviews with rehabilitation staff, it was concluded that data saturation was achieved. MAXQDA (version 10) was used for data management.

### Quantitative data and analysis

To assess the effectiveness of the falls prevention program, the research team requested the patient safety unit to provide information on patients’ fall history both four months prior to and during the study. Fall prevention procedures in this hospital typically involve assessing patient fall risk using the Morse scale, completing an incident checklist when a fall occurs, and conducting formal post-fall huddles or root cause analysis (RCA) during monthly staff meetings in the inpatient rehabilitation setting. Discussions during these meetings focus on reasons for the fall, whether the patient was identified as high risk, and whether appropriate interventions were implemented. To evaluate the implementation of fall prevention measures in the SCI unit, the Fall Prevention Measures Checklist developed by the World Health Organization and Ministry of Health and Medical Education was utilized [25]. To establish the content validity of the fall prevention measures checklist among a group of patients admitted to the rehabilitation hospital, additional items were incorporated into the checklist and modifications were made based on the findings from the qualitative stage of the study and the results of the literature review. The checklist comprised 26 items addressing three dimensions: monitoring and care (11 items), patient education (7 items), and environmental safety (8 items). Each item was scored on a scale of 0 to 2, with a total score of 39 indicating the boundary between favorable and unfavorable performance. The content validity index (CVI) was evaluated using the Waltz and Basel content validity index approach. For each item, the criterion of "cross-cultural relevance" was assessed on a 4-point Likert scale (1 = irrelevant, 2 = somewhat relevant, 3 = relevant but needs modification, 4 = completely relevant). Subsequently, the items with the highest ratings (3 or 4) were divided by the total number of experts. A CVI of 0.79 or higher was considered acceptable [26]. To guarantee the reliability of the checklist, two evaluators independently assessed the SCI unit over four consecutive months using this tool. Inter-rater reliability was determined using the weighted kappa coefficient, indicating good agreement between the two raters (κ <0.85 across all items).

The severity of injuries resulting from falls was categorized as mild, moderate, severe, and death, in accordance with WHO definition.

#### Mild

Asymptomatic or mild symptoms; clinical or diagnostic observations only; intervention not required.

#### Moderate

Minimal, local, or noninvasive intervention indicated; affecting age-appropriate instrumental Activities of Daily Living (ADL).

#### Severe

Medically significant but not immediately life-threatening; hospitalization or prolongation of hospitalization necessary; potentially disabling.

#### Death

Fatality related to adverse events (AEs) [27, 28].

Quantitative data were analyzed using SPSS (version 22) and using descriptive (prevalence, mean, and standard deviation) and inferential statistics (paired-t-test and Chi square tests). P-value < 0.05 was regarded as statistically significant.

### Trustworthiness

To ensure the trustworthiness of the data, the study addressed criteria such as credibility, dependability, confirmability, and transferability proposed by Lincoln and Guba [29]. To ensure credibility and dependability, the data was thoroughly engaged with over an extended period. The initial coding of interviews underwent member check, where participants verified the accuracy of the codes. Additionally, three faculty members familiar with qualitative research conducted peer review and agreed on interpretations of the codes and categories. confirmability was also ensured by documenting all the stages of the study to provide an opportunity for others to replicate research-related activities. Finally, transferability was established through sampling with maximum diversity in terms of age, professional experience, and education level of the participants.

### Ethics approval and consent to participate

This study was approved by the Ethics Committee of University of Social Welfare and Rehabilitation Sciences on May 20, 2020 (IR.USWR.REC.1399.058).Obtaining permission from the authorities of Rafidah Rehabilitation Hospital, fully explaining the objectives and nature of the research, and assuring the confidentiality of patients’ personal information were among the ethical considerations that were followed. They were also informed about the voluntary participation or withdraw from the study. Written informed consent was obtained from all participants.

## Results

In interviews and focus group discussions, 19 staff, including physicians and nurses at different job categories, physiotherapists, occupational therapists, and speech therapists, were recruited. In terms of occupation status and position of the participants, nurses at different job categories (n = 8), rehabilitation team (n = 3, occupational health specialist, occupational therapist, physiotherapist), a group of senior managers (n = 4, the head of the hospital, Deputy of treatment, internal manager, matron), and 3 supervisors (health education supervisor, patient health education supervisor, clinical supervisor) were present. The maximum and minimum professional experience was 30 years and 2 years, respectively. Among the participants, most of them were female and married (Table 1).

**Table 1:** The participants’ demographic characteristics.

Variable		N(%)
Age group (Year)	22–29	4(21.1)
	30–39	7(36.8)
	40–49	7(36.8)
	≥ 50	1(5.3)
Gender	Male	6(31.6)
	Female	13(68.4)
Education level	Bachelor	4(21.1)
	Master	9(47.3)
	PhD	4(21.1)
	MD	2(10.5)
Occupation status	Nurse	13(68.4)
	Physician	3(15.7)
	Therapist	3(15.7)
Professional experience (year)	>10	8(42.1)
	10–20	7(36.8)
	20–30	4(21.1)

Based on the results, before the interventions, 19 falls (29.23%) occurred in the SCI ward. Falls were most common among patients aged fifty years or older (12.3%) (P = 0.026). Men (10, 15.38%) were more likely to experience falls during the study period than women (9, 13.84%) (P = 0.01). Most fall events occurred in the morning (11, 57.89%) (Table 2). The majority of identified adverse events were of mild severity (15, 78.94%), with a smaller portion being moderate (3, 15.78%) and severe (1, 5.26%). No fatal falls were identified during the study period. Falls were more common when patients independently performed their activities and mostly occurred in patient rooms or during transfer to bed or wheelchair.

**Table 2:** The relationship between falls and age group and gender in SCI patients.

Variable		N(%)	P value
**Age group (Year)**	22–29	5(7.69)	0.02*
	30–39	4(6.15)	
	40–49	3(4.61)	
	≥ 50	8(12.3)	
**Gender**	Male	10(15.38)	0.01*
	Female	9(13.84)	

* Chi-squared test

### Problem identification

After analyzing the data from individual interviews and focus group discussions, finally, two main problems were identified (Table 3).

**Table 3:** Categories and sub-categories obtained in the qualitative phase.

Categories	Sub-categories
Lack of patients and caregivers’ knowledge and participation	Unfamiliarity of patients and their families with their duties
	Deficiency in patient education methods
Shortcomings in fall prevention policies	Failure to identify high-risk patients
	Negligence of high -risk patients
	Inefficient interprofessional interactions

#### 1) Lack of patients’ and caregivers’ knowledge and participation

Some participants stated that the training provided to patients and caregivers was sometimes unclear, hindering their ability to provide safe care. They also highlighted several factors contributing to this issue, including inadequate comprehensiveness of the patient training material regarding safety compliance, nurses’ heavy workload leading to time constraints, incomplete understanding of the training content, absence of feedback, inadequacy of the current training approach, and patients’ lack of cooperation and attentiveness to their responsibilities. These factors collectively contribute to the lack of knowledge and participation among patients and caregivers.

*“Well*, *some patients don’t cooperate with the nurses*. *The patient doesn’t pay attention*, *engages in risky behavior*, *and may be at risk of falling*

*(participant No*.*18)*.

*"…Patient education is done but incompletely*. *Because feedback is not taken from the patient to make sure that the patient has understood the education or not*…*"*

*(Participant No*. *1)*.

#### 2) Deficiencies in fall prevention policies/procedures

The implementation of evidence-based fall prevention strategies in rehabilitation centers is constrained. Moreover, there exists no conclusive evidence regarding the effectiveness of fall prevention programs in hospitals. This category encompasses two subcategories: failure to identify high-risk patients and inadequate sensitivity towards high-risk patients.

*A) Failure to identify high-risk patients*. Many nurses expressed doubts regarding the Morse fall prevention scale’s ability to identify high-risk patients with spinal cord injury (SCI). Primarily utilized in acute care hospitals, this scale lacks specificity and suitability for rehabilitation patients. Its efficacy in predicting fall risk among rehabilitation patients is subpar. Consequently, its usage can impede the effective implementation of fall prevention measures and diminish sensitivity towards genuine high-risk patients.

*"For example*, *the Morse fall prevention scale gives a zero score to the item of using a wheelchair*, *which is related to the ambulatory aids domain*. *While most rehabilitation patients depend on wheelchairs and may fall during transfer*.*"*

*(Participant No*. *11)*.

*B) Insensitivity to high-risk patients*. The participants referred to the use of yellow wristbands for all patients and reduced sensitivity to real high-risk patients as factors that threatened patient safety.

*"Here*, *all our patients are considered high-risk and a yellow wristband is used for every patient*. *Since all patients have a yellow wristband*, *sensitivity to real patients who are at high risk of falling is reduced and the patient may fall*.*"*

*(Participant No*.*7)*

*C) Inefficient interprofessional interactions*. The participants cited lack of coordination in care provision, irregularity in patient transport, and inadequate collaboration between nurses and the rehabilitation team as examples of weak teamwork and limited interprofessional interactions.

" *When a patient is transferred from the SCI unit to our unit for rehabilitation*, *we only have access to the patient and are unable to communicate with the unit nurses to inquire about the patient’s clinical status…*.*"*

*(Participant No*.*14)*.

### Planning and action

At this stage, the strategies proposed by participants, combined with insights from the literature review on developing fall prevention programs in the SCI unit, led to the identification of four main categories: education and training, risk assessment, monitoring and surveillance, and a multidisciplinary approach (Table 4).

**Table 4:** Suggested fall prevention strategies in the SCI unit.

Objectives	Main categories	Action plan
Improving fall prevention procedures/policies	Risk assessment	Identifying high -risk patients and distinguishing them from other patients
	Monitoring and surveillance	Hourly rounding
	Multidisciplinary approach	Enhancement of interprofessional interaction
Promotion of patients’ and caregivers’ knowledge and participation	Education and training	Education through conducting workshops
		Preparation of reminders and booklets
		Group education sessions with patients with similar health problems

#### 1) Improving fall prevention procedures/policies

According to the participant’s point of view, paying attention and revising fall prevention policies was an effective strategy to improve patient safety. The participants stated that strategies, such as the use of specific fall risk assessment tools and hourly assessment of patient’s condition can help to identify high-risk patients and make an appropriate decision regarding medical care.

*A) Identification of high-risk patients*. The tool identified the predictors or risk factors of falling as the diagnoses, Functional Independence Measure (FIM) items, and history of fall. Psychometric analysis and localization were conducted to determine the content validities of the designed scale using Content Validity Index (CVI). The inter-rater reliability was evaluated through the Kappa Coefficient (Κ). Scale-level CVI was 94% and Cohen’s kappa coefficient was 70–92%. Then, it was decided to distinguish high-risk patients from other patients by using a yellow wristband.

*B) Hourly rounding*. Several participants highlighted the effectiveness of hourly rounding in reducing fall rates and enhancing the monitoring of patients’ clinical conditions. Following the education of nurses and nursing assistants on the program’s purpose and expectations, the hourly rounding process was implemented for a period of four months. During this time, patients were checked every two hours for pain level, body position, room furniture arrangement, bathroom or toilet needs, potential fall risks, bed rail positioning, and other environmental factors. Following each rounding visit, the information was documented in the patient’s electronic health record. Patient satisfaction with care was also assessed upon discharge.

*C) Enhancement of interprofessional interaction*. According to the participants, interprofessional collaboration, during which two or more professions learn from and about each other, can foster safety, teamwork, and a patient-centered care climate. A continuous education program focusing on communication skills was designed and implemented to develop professional scientific communication between nurses and rehabilitation team members. In this program, it was decided that nurse unit managers hold monthly meetings to investigate the root causes of a patient falling.

#### 2) Increase in patients’ and caregivers’ awareness and participation

As per the participants, educating patients and caregivers on safety standards, their physical condition, and the risks and consequences of falling can familiarize them with fall prevention strategies, enhancing psychological capacities and motivation. Some participants recommended group education sessions to facilitate shared experiences, mutual learning, and increased patient participation. To enhance patients’ knowledge and awareness, group education sessions were organized with patients sharing similar health concerns. Additionally, reminders about fall prevention strategies, such as posters, pamphlets, and educational booklets, were disseminated. Furthermore, in collaboration with the nursing office, a nurse was designated to provide education to patients and their companions on these matters.

### Observation and evaluation of the program

To assess the effectiveness of the implemented programs, a mixed-method evaluation approach was employed. Qualitative evaluation involved observation, while quantitative evaluation utilized a falls prevention measures checklist. Additionally, the number of patient falls was compared before and after the implementation of the program.

The researcher assessed fall prevention measures in the SCI unit by utilizing a fall prevention measures checklist. This checklist was employed to evaluate nurses’ performance in the SCI unit both before and after interventions. Subsequently, the mean score of each checklist item was computed. The findings indicated that, prior to the intervention, the mean and standard deviation score for patients’ monitoring and care was 7.07 ± 5.5, patient education was 4 ± 5.65, and environmental safety was 4.5 ± 6.36, all of which were at low levels. Following the intervention, the mean scores for patients’ monitoring and care rose to 21 ±.000, patient education to 7 ± 8.89, and environmental safety to 5.5 ± 7.7, demonstrating a significant improvement as per the paired-sample t-test (P<0.001). In essence, adherence to fall prevention measures increased subsequent to the implementation of the intervention (Table 5).

**Table 5:** Comparison of the mean and standard deviation of fall prevention measures checklist before and after the intervention.

Variables	Before the interventions	After the interventions	t	*P*-value
Patients’ monitoring and care	5.5 ± 7.07	21 ± .000	3.2	P<0/001*
Patient education	4 ± 5.65	7 ± 9.8 .89	0.27	P<0/001*
Safety of environment	4.5 ± 6.36	5.5 ± 7.7	0.1	P<0/001*

*Paired T-Test

Having investigated the fall reports in the SCI unit between August 2021 and June 2022, it was found that there were 10 fall cases among 80 admitted patients during 4 months before the intervention and 2 fall cases among 80 admitted patients during 4 months after the intervention. These results showed that the occurrence of falls significantly decreased after the intervention(P = 0/02).

#### Re-feedback

During the re-feedback stage, improvements to the fall prevention program were made based on findings from observations, document reviews, and a reassessment of the program’s effectiveness, weaknesses, and strengths. Additionally, problems related to program implementation were identified from the perspectives of healthcare and rehabilitation teams. Subsequently, decisions were made regarding strategies and methods to enhance practices in this action research study. Following the program’s implementation, weaknesses were identified during the evaluation stage, such as insufficient attention to staff training in rehabilitation units, staff reluctance to report falls in rehabilitation units, and the inability to conduct in-person workshops for all staff due to the COVID-19 pandemic. Addressing these issues and rectifying weaknesses are essential for the continuation of the program.

## Discussion

This study revealed that the designed program, which focused on enhancing interprofessional interaction, pinpointing high-risk patients unique to the inpatient rehabilitation hospital setting, and conducting educational workshops for patients and caregivers, facilitated the adoption of essential and pertinent measures to prevent and diminish patient falls in the SCI unit.

The majority of recorded falls occurred in the morning, potentially attributed to the higher workload of the hospital during this time compared to the evening or night shifts. Our findings consistent with previous research. For instance, Saverino et al. reported 40 fall events among 320 patients hospitalized for rehabilitation over a seven-month period, with 55% of the patients being male [30]. Additionally, Mirapeix et al. highlighted falls as the most prevalent adverse events (AEs) in Post-Acute Rehabilitation Care, especially among individuals aged 75 years and above [31]. Tsur et al. reported that 65% of fall events (n = 52) occurred during the daytime, 55% (n = 44) during transfers, 65% (n = 52) within the patient’s room, and only 18.8% (n = 15) in the toilet/bathroom. Given that a significant proportion of patients in rehabilitation hospitals experience cognitive and sensorimotor challenges, diligent supervision and monitoring by dedicated rehabilitation staff can help decrease the fall rate [32, 33].

In this study, more than half of the falls were categorized as mild in severity. Most patients who experienced falls recovered without permanent disability, and the harm caused by falls was predominantly low in severity. One potential explanation is that hospital staff may have felt unsupported in reporting serious falls, fearing blame from their ward for error reporting, thus reducing the likelihood of voluntary reporting of such incidents. Employing combined methods such as offering constructive feedback on errors, avoiding punitive measures, and involving patients as partners in identifying Adverse Events (AEs) can contribute to enhancing patient safety [34].

Based on the findings of this study, participants expressed a preference for replacing the current fall risk assessment tool with one that could specifically evaluate the limitations and risk factors for falls in rehabilitation patients. Most of the current fall risk assessment tools utilized in rehabilitation hospitals, such as the Morse Fall Scale, Hendrich II Fall Risk Model, Downton, and STRATIFY instrument, were originally developed and evaluated in acute care settings [35]. Several studies have examined these risk assessment tools. They found that although these tools were suitable for measuring the risk of falling in general, their use in rehabilitation hospitals could not be beneficial given that these tools showed high sensitivity to rehabilitation patients and estimated high risk of falling in most of these patients [35–39]. To address this issue, the Persian version of the Fall Risk Assessment Scale (FRAS) was developed, with established reliability and validity for patients admitted to rehabilitation hospitals, offering a tailored solution to the challenges posed by existing assessment tools in this specific healthcare setting.

High-risk patients were then identified and differentiated from other patients using yellow wristbands. Loo et al., (2019) also used color-coded wristbands (CCWs) to inform rehabilitation staff about the specific conditions of patients in the neurology unit. CCWs remind staff and caregivers to provide these patients with a safer care environment [40]. Distinguishing high-risk patients allows for the use of appropriate fall-prevention interventions in the target group.

Another subcategory of the promotion of fall prevention policies was hourly rounding. Hourly rounding is an important measure that nurses can resort to in order to improve patient safety and reduce falls by 50% in hospitals [41]. Gathering information about patients in their environment, assessing risk factors, and developing an individualized plan of care were all part of this intervention. Hourly rounding promotes patient-centered safety interventions and improves communication between staff and patients in a rehabilitation setting. Creating and maintaining a safe environment to prevent fall means being aware of the patient’s surroundings, ensuring effective communication among team members, reporting and sharing ideas about falls, and using the nurse’s clinical judgment and critical thinking to predict the likelihood of falls [42].

Another factor requested by the participants in this study was the development of interprofessional communication and collaborative care. Rehabilitation specialists often work as a team, but the autonomy of some disciplines and their lack of coordination in providing care and rehabilitation services create a communication gap between them, and as a result, hurts patients [43]. Franz et al., (2018) showed that the quality of patient care could be improved through interprofessional communication among rehabilitation team members and members’ consultation while making decisions [44]. Singh et al., (2020) also revealed communication amongst staff was an important factor in reducing fall in inpatient SCI rehabilitation. In fact, one discipline alone cannot identify all the risk factors because patient fall is a complex multifactorial phenomenon. The effective approach is to create a safe environment for the patient by reinforcing interprofessional communication in a rehabilitation environment and reporting and sharing ideas about fall prevention.

In response to the lack of knowledge and participation among patients and caregivers, training on fall prevention was offered to empower these groups. A study conducted by Heng et al. (2020) found that interventions including face-to-face education for patients regarding fall risks, the distribution of brochures and pamphlets, and the adjustment of hospital policies were effective in preventing patient falls [45]. In China, Cheng et al. (2018) also found that training and involvement of patients and caregivers in fall prevention programs during patients’ rehabilitation programs reduced the incidence of falls [46]. In this study, opportunities were also given for patients with similar health issues to communicate, allowing those with negative beliefs and attitudes to voice their concerns, thus enhancing their motivation to continue treatment and adhere to patient safety standards. Engaging patients and caregivers in fall prevention strategies, addressing perceived barriers, and providing supportive resources seem to influence clients’ health beliefs positively and increase awareness of fall risks. To accomplish this, fall prevention training should involve not only nursing staff and rehabilitation teams but also patients and their companions. These initiatives facilitate the implementation of fall prevention strategies and contribute to a reduction in falls [47].

In this action research, the impact of the fall prevention program was assessed during the observation and reflection stages. The findings indicated a significant decrease in the incidence of patient falls and an increase in nurses’ confidence and job satisfaction, thanks to the collective efforts of all stakeholders. Jitramontree et al. (2015) successfully implemented falls prevention programs in their action study, which encompassed fall risk assessments, balance exercises, muscle strengthening, cane walking training, educational booklets, and environmental safety assessments. Through staff participation and collaboration, they effectively reduced falls among the elderly [9]. Likewise, the current study, employing a participatory process involving all stakeholders, witnessed an improvement in participants’ motivation and commitment to instigate change, resulting in the enhanced efficacy of the fall prevention program.

### Study limitations

Given that this study was conducted exclusively at Rofaydeh Rehabilitation Hospital, the sole facility of its kind in the country, caution should be exercised when attempting to generalize the findings to other rehabilitation centers.

## Conclusion

The study showcases that a multifaceted intervention can significantly decrease falls and injuries even with a lower dosage. Sustaining this program necessitates policy formulation and meticulous planning within the healthcare system, ultimately enhancing patient safety and elevating the standard of care. Future research is recommended to assess this program across various units within rehabilitation hospitals, employing both quantitative and qualitative methodologies.
